# Health literacy and self-medication among older adults in rural Thailand: understanding inequities in a digital era

**DOI:** 10.1080/16549716.2025.2572008

**Published:** 2025-10-15

**Authors:** Tharinee Srisaknok, Chanuttha Ploylearmsang, Adisorn Wongkongdech, Souksathaphone Chanthamath, Ranee Wongkongdech

**Affiliations:** a Faculty of Pharmacy, Mahasarakham University, Maha Sarakham, Thailand; b International and National Collaborative Network and Innovation for Community Health Development Research Unit (INCNI-CHD), Thailand; c Social and Administrative Pharmacy Group, Faculty of Pharmacy, Mahasarakham University, Maha Sarakham, Thailand; d Faculty of Public Health, Mahasarakham University, Maha Sarakham, Thailand; e Public Health and Environmental Policy in Southeast Asia Research Cluster (PHEP SEA Thailand); f Department of Internal Medicine, Khammouane Provincial Hospital, Thakhek District, Khammouane Province, Lao PDR; g Faculty of Medicine, Mahasarakham University, Maha Sarakham, Thailand

**Keywords:** aging population, digital literacy, health-seeking behavior, medication safety, Thailand

## Abstract

**Background:**

Inappropriate self-medication and limited health literacy are pressing challenges that contribute to poor health outcomes among older adults. In rural, low-resource settings, these issues are worsened by digital exclusion, which restricts access to reliable health information and informed decision-making.

**Objective:**

To assess the prevalence and determinants of inappropriate medication and health product (M&HP) use among older adults in rural Thailand, reflecting wider disparities in Southeast Asia.

**Methods:**

A cross-sectional survey was conducted among 1,461 older adults aged ≥60 years, selected through stratified multi-stage sampling from Thailand’s Health Regions 7–10, which encompass 20 northeastern provinces. Structured interviews explored health-seeking behavior, digital literacy, and M&HP use. Inappropriate use was defined as the use of two or more items without professional consultation, as per national guidelines. Descriptive and multivariable logistic regression analyses were performed.

**Results:**

Of participants, 84.5% reported using M&HPs without professional consultation, and only 14.0% were proficient in accessing health information via smartphones. Inappropriate use was significantly associated with rural residence (aOR = 6.33; 95% CI: 2.19–18.31), low education, chronic illness, unemployment, digital illiteracy, and financial hardship (*p* < 0.05).

**Conclusion:**

Digital and social exclusion strongly influence unsafe self-medication practices among older adults. Improving digital health literacy and access to credible information is crucial for enhancing medication safety. Thailand’s experience may offer valuable guidance for developing inclusive aging strategies in other low- and middle-income countries.

## Paper context

● **Main findings:** Older adults in rural Thailand demonstrated high rates of inappropriate medication and health product (M&HP) use, primarily influenced by limited health literacy, digital exclusion, and socioeconomic vulnerability. Key predictors included rural residence, low educational attainment, unemployment, and limited digital literacy.

● **Added knowledge:** This study expands the current understanding of how structural inequities and digital barriers interact to shape health behaviors in aging populations within low-resource settings. It is one of the few large-scale studies in Thailand quantifying the determinants of inappropriate self-medication among older adults.

● **Global health impact on policy and action:** Findings offer critical insights for public health policies targeting safe medication use, digital inclusion, and health literacy interventions among older adults in low- and middle-income countries (LMICs). Policymakers can utilize these insights to design inclusive programs that reduce health disparities and support aging in place in underserved communities.

## Background

The global population is undergoing a profound demographic shift, with the number of individuals aged 60 years and older surpassing one billion in 2019. This figure is projected to reach 1.4 billion by 2030 and 2.1 billion by 2050, marking a rapid expansion of the aging population worldwide [1]. Thailand mirrors this trend, with over 12 million older adults – approximately 18% of the national population – a number expected to rise sharply in the coming decades [2]. Northeastern Thailand is a critical focus in this demographic landscape, hosting nearly 19% of the country’s older population, and ranking as one of the most rapidly aging regions in Southeast Asia, second only to Singapore [3,4]. By 2037, the number of Thai adults aged 80 and older is projected to double, with nearly 5% of older adults already requiring assistance with daily living activities [5].

Older adults often live with multiple chronic conditions and require long-term care. However, aging-related declines in cognitive and sensory function, alongside social isolation and environmental barriers, can hinder their ability to manage their health independently [6,7]. In Thailand, national surveys have reported widespread limitations in health literacy among older adults, with many engaging in risky health behaviors such as inappropriate self-medication and use of unregulated health products [8]. These challenges are particularly acute in the northeastern region, where critical health literacy skills, including evaluating medication labels and product reliability, remain notably low [9,10].

In this study, health literacy refers to the ability to access, understand, and use health information. In contrast, digital literacy involves the capacity to use digital tools to find and apply such information, both of which are essential for safe medication and health product use among older adults.

To address these issues, Thailand has implemented long-term care (LTC) policies and community-based support systems designed to promote healthy and active aging [11,12]. However, shifting social dynamics – such as declining family size, lower fertility rates, and rural-to-urban migration – have eroded the traditional caregiving roles of families [13]. In response, the government has trained community-based caregivers and volunteers to provide essential support for dependent older adults [14].

Internationally, the World Health Organization (WHO) advocates for the rational use of medicines, encompassing appropriate prescribing, dispensing, and consumption practices [15]. Thailand has aligned with these principles through initiatives such as Rational Drug Use (RDU) policies, digital health promotion reforms, and the ‘Smart Elderly Citizen’ program, which aims to improve informed decision-making in the use of M&HPs [16,17].

Despite these efforts, challenges remain. Self-medication without professional consultation, reliance on unverified information sources, and exposure to misleading health product advertising persist among older adults [6]. These behaviors are exacerbated by limited digital skills and increased access to unregulated online markets – factors particularly concerning for older adults with cognitive or literacy limitations [8]. The inappropriate use of M&HPs not only compromises individual health and safety but also contributes to household financial burdens and increases the strain on the healthcare system [18].

This issue is further compounded by the digital divide, particularly in rural settings where access to technology and digital literacy is low. According to the Health Behavior and Health Literacy Framework [5], individuals with limited access to digital tools and low health literacy are more likely to make uninformed decisions, engage in self-care risks, and delay professional consultation.

This study adopts this framework to examine how health and digital literacy influence older adults’ behaviors regarding the use of M&HPs. The framework emphasizes the interplay between individual capabilities and structural barriers, providing a lens through which to interpret patterns of inappropriate M&HP use.

In this context, Northeastern Thailand – where aging, poverty, and disparities in healthcare access converge – presents a compelling case for research. This study aims to examine patterns of medication and health product (M&HP) use among older adults in this region and to identify key factors associated with inappropriate use. The findings can inform context-sensitive strategies to enhance medication safety, promote digital and health literacy, and reduce inequities in aging populations across low- and middle-income countries (LMICs).

## Methods

### Study design and setting

This study employed a cross-sectional survey design. Data were collected through structured interviews between April 1 and 30 September 2021, from older adults (aged ≥60 years) residing in rural communities across the I-saan region of Northeastern Thailand.

Study sites were proportionally selected based on population size across Health Regions 7 to 10—administrative zones established by the Ministry of Public Health to support regional healthcare planning, which covers 20 provinces in Northeastern Thailand, ensuring representative sampling. A multi-stage random sampling approach was used to select elderly schools – institutions promoting lifelong learning and health promotion among older adults [19]. Elderly schools were randomly sampled from official provincial lists, yielding four schools in Maha Sarakham Province (Region 7; 354 participants), three schools in Loei Province (Region 8; 342 participants), three schools in Nakhon Ratchasima Province (Region 9; 478 participants), and three schools in Sisaket Province (Region 10; 287 participants).

### Participants and recruitment

An initial sample size of 1,537 participants was calculated using Cochran’s formula (Cochran, 1977, as cited in [19], based on a 95% confidence level and an estimated variance of 8.18 [20]. To account for incomplete responses and potential attrition, the sample size was increased by 20%, resulting in a target of 1,922 participants.

Eligible participants were community-dwelling adults aged 60 years and older, regardless of chronic condition status, who were fully conscious, able to communicate in Thai, and willing to provide informed consent. Individuals who were hospitalized during data collection, required urgent treatment outside the study area, or were unable to cooperate were excluded. After excluding incomplete responses, a final sample of 1,461 participants was included in the analysis. After excluding incomplete responses, a final sample of 1,461 participants was included in the analysis. Participants with missing data on key variables were excluded, and a complete case analysis was performed without imputation.

### Questionnaire development

A structured questionnaire was developed to assess health behaviors and the use of M&HPs (M&HPs) among older adults. Five experts in public health, pharmacology, and geriatric care evaluated its content validity, with all items achieving an Item-Objective Congruence (IOC) score greater than 0.5. Based on expert feedback, the questionnaire underwent three rounds of revision. A pilot study with 30 older adults was conducted to assess feasibility and reliability. Cronbach’s alpha coefficient was 0.97, indicating excellent internal consistency.

### Questionnaire scoring

The questionnaire consisted of 28 items: 12 related to socio-demographics, seven to general health behaviors when ill, and nine to M&HP use behaviors. The 9-item behavior section was scored using a three-point Likert scale (Always = 3, Sometimes = 1, Never = 0) to assess practices over the past six months. Items were scored accordingly, with items worded positively or negatively. Composite scores were classified as low (1.00–1.50), moderate (1.51–2.50), or high (2.51–3.00). This cut-off point was guided by prior research emphasizing the clustering of multiple health risk behaviors, where engaging in two or more risky behaviors has been linked to increased adverse health outcomes [5]. Applying this threshold enhances the interpretability of behavioral risk patterns in public health research, particularly among older adults [21].

### Data collection

All field researchers, including 16 community-based assistants and four provincial coordinators, participated in a two-day standardized training program led by the principal investigators and geriatric health specialists. The training covered interview techniques, informed consent procedures, digital literacy assessment, and COVID-19 safety protocols to ensure consistency across all sites.

Structured interviews were conducted primarily at participants’ homes. For those unable to participate in person, interviews were adapted to telephone or online platforms, with caregiver assistance provided as needed. Written or verbal informed consent was obtained from all participants before data collection.

### Ethical considerations

The study protocol was reviewed and approved by the Mahasarakham University Human Research Ethics Committee (approval number 077/2564, date of approval: 18 February 2021). All procedures were conducted by the Declaration of Helsinki and relevant local regulations. The study’s objectives, voluntary participation, confidentiality assurances, and consent procedures were explained verbally, in writing, or via telephone, depending on the mode of data collection. Participants received a small token of appreciation after completing the survey.

### Operational definitions

- **Inappropriate M&HP Use**: Self-use of medications or health products without professional consultation, relying on non-professional sources, or failing to verify product reliability. Participants who reported two or more such behaviors within the past six months were classified as inappropriate users.
- **Health Products**: Non-prescription items used for health maintenance or improvement, including dietary supplements, herbal remedies, traditional medicines, topical balms, vitamins, and wellness items. These are often self-selected and used without professional consultation.
- **Low Health Literacy**: Difficulty accessing, understanding, evaluating, or applying health information. Health literacy was measured using a validated tool and categorized into tertiles based on score distribution. Participants in the lowest tertile were classified as having low health literacy.
- **Digital Literacy**: The ability to effectively use digital tools (e.g. smartphones, internet) to seek, understand, and use health information. Participants who regularly used digital devices for health-related purposes were considered digitally literate.
- **Pensioner**: A retired individual receiving a regular pension or government-provided retirement support, commonly seen among older adults no longer engaged in paid employment.
- **Health Seekers**: Individuals who actively seek out health-related information or services, whether through traditional healthcare providers, community health resources, or digital platforms. This behavior reflects proactive engagement in personal health management.

### Primary outcome variables

- Inappropriate M&HP Use: Binary (appropriate vs. inappropriate use) based on operational definition.
- Health Literacy Level: Categorical (low, moderate, high) according to health literacy scores.
- Digital Literacy Status: Binary variable (digitally literate vs. digitally illiterate) based on self-reported device use for health information-seeking.

### Statistical analysis

Descriptive statistics summarize participants’ socio-demographic characteristics, health behaviors, health literacy levels, digital literacy status, and medication and health product (M&HP) use patterns. Frequencies and percentages were reported for categorical variables, while means and standard deviations were reported for continuous variables.

Associations between socio-demographic factors and inappropriate M&HP use were initially assessed using chi-square tests for categorical variables and independent t-tests for continuous variables. Binary logistic regression was then conducted to identify independent predictors of inappropriate M&HP use. Adjusted odds ratios (aORs) with 95% confidence intervals (CIs) were calculated. The multivariable logistic regression model included variables with a p-value less than .20 in univariate analyses. Statistical significance was defined as *p* < .05. Data were entered, verified, anonymized, and analyzed using RStudio (Version 2022.02.1 + 461, ‘Prairie Trillium’) [22] with descriptive and inferential statistics.

## Results

### Participant characteristics

Among the 1,461 older adult participants, the mean age was 68.60 years (SD = 5.99), and 59.75% were female. Most participants resided in rural areas (97.31%), had educational attainment not exceeding grade 6 (93.98%), were living with a spouse (62.90%), and were primarily farmers (61.53%). Only 27.52% reported sufficient income, and 78.64% had at least one chronic condition. Regarding digital literacy, 44.56% were unable to use smartphones, and only 14.03% reported being proficient in using smartphones to access health information. Additionally, 39.22% reported receiving support from medical personnel, and 91.10% expressed a need to improve their literacy in medication and health product usage (see Table 1).

**Table 1.** Participants’ demographics (*N* = 1,461).

Personal factors	Number	Percentage
1. Gender		
Male	588	40.25
Female	873	59.75
2. Age $\bar{x}=68.60$ (S.D. = 5.99), Min = 60 years, Max = 87 years		
3. Place of residence		
1) urban	43	2.69
2) rural	1,556	97.31
4. Highest education level		
1) not exceed grade 6	1,373	93.98
2) above grade 6 (primary school)	88	6.02
5. Marital status		
1) Single	48	3.29
2) Living with spouse	919	62.90
3) Divorced or widowed	494	33.81
6. Caregiver		
1) do not have caregivers	272	18.62
2) family members	1189	81.38
7. Occupation		
1) Farmers	899	61.53
2) Pensioner	30	2.05
3) Unemploying	532	36.41
8. Have enough money	440	27.52
9. Underlying disease	1149	78.64
10. Have a smartphone(s) and can use it to find health information		
on the internet		
1) proficiency	205	14.03
2) not proficiency	605	41.41
3) cannot use	651	44.56
11. Have support from medical personnel to provide help/advice on medications and health product usage.	573	39.22
12. Need greater literacy in the usage of medicines and health products	1331	91.10

Note: This table presents the demographic characteristics of 1,461 older adults, including age, gender, and education level.

### Health-seeking behaviors

Among the participants, 84.5% reported using medications or health products without professional consultation. Supplements were the most commonly used products, often consumed for general health maintenance rather than managing specific illnesses. Health information was predominantly obtained from informal sources, including media (49.2%), family members (37.6%), and peers (42.3%). While some participants sought care from hospitals or clinics when ill, a considerable proportion relied on alternative sources such as grocery stores (32.1%) or advice from acquaintances (28.7%). Access to professional pharmacy services and digital health resources remained limited (see Table 2).

**Table 2.** Health care-seeking behaviors of the Thai elderly (*N* = 1,461).

Personal factors	N	%
**When symptoms of illness occur**		
**1. How to Seek Information about Health, medicine, or health products**		
1) Search for information by her/himself	612	41.89
2) Often ask others for information	290	19.85
3) Do not seek additional information	559	38.26
**2. How to manage and treat illness (multiple answers allowed)**		
1) Consult a current physician at the hospital	1404	45.64
2) Consult a current physician at the clinic	581	18.89
3) Buy medicine from a pharmacy with a pharmacist on duty	0	0.00
4) Buy medicine from a pharmacy (not sure if there is a pharmacist)	95	3.09
5) Buy from a local grocery store	319	10.37
6) Buy from media advertisements such as T.V., radio or online	270	8.78
7) Seek advice/medicine from someone with similar symptoms	311	10.11
8) Have family members arrange it	96	3.12
**3. Receive Health Information about Medicine or Health Products from**		
1) Healthcare professionals	1082	24.66
2) Village health volunteers (VHVs)	599	13.65
3) Community leaders	151	3.44
4) Family members, relatives, siblings	504	11.49
5) Neighbors	239	5.45
6) Television	624	14.22
7) Radio	478	10.90
8) Brochures, books, leaflets, magazines	147	3.35
9) Social media and online platforms such as Line, Facebook or YouTube	563	12.83
**4. Experience in Choosing Medicine and Health Products Without consulting medical personnel**	1234	84.46
**5. Reasons for choosing medicine or health products**		
1) Treatment of illness	660	48.73
2) Health maintenance	801	51.27
**6. Types of medication or health products used** (Multiple answers allowed)		
1) Medication	550	31.65
2) Health supplements	920	52.93
3) Medical devices	268	15.42
**7. Reasons for Choosing Medicine and Health Products** (Multiple answers allowed)		
1) Personal decision	598	36.87
2) Recommended by others	854	52.65
3) Given by others	50	3.08
4) Advertising	120	7.40

Note: The number of respondents for each item may vary due to non-response or item applicability. Analyses were conducted based on available data.

### Medication and health product (M&HP) use behaviors

Risk behaviors associated with M&HP use were commonly observed. Only 40.3% of participants regularly read drug labels and 53.9% verified product registration numbers. Although 68.2% of participants used dietary supplements, these were often chosen based on non-professional recommendations. Consultation with healthcare providers occurred more frequently when participants experienced abnormal symptoms (58.4%). However, sharing unverified health information and accepting medications or products from others were prevalent practices among the study population (see Table 3).

**Table 3.** Usage of medicine & health products behaviors of Thai elderly (*N* = 1,461).

	Always (3 pts.) N (%)	Sometime (1 pts.) N (%)	Never (0) N (%)	$\overset{ˉ}{x}$ ± S.D.	M&HP Usage Level
1. Seek information from reliable sources before making a purchase	181 (12.39)	931 (63.72)	349 (23.89)	1.01 ± 0.86	Low
2. Read the drug label before usage	413 (28.27)	763 (52.22)	285 (19.51)	1.37 ± 1.09	Low
3. Verify information from M&HP advertisements before making a purchase	330 (22.59)	431 (29.50)	700 (47.91)	0.97 ± 1.18	Low
4. Consult a doctor or pharmacist promptly if experiencing abnormal symptoms	428 (29.30)	959 (65.64)	74 (5.07)	1.54 ± 0.97	Moderate
5. Share or forward information without verifying quality	180 (12.32)	426 (29.16)	855 (58.52)	0.66 ± 0.98	Low
6. Verify information before deciding to purchase or use medications or products	399 (27.31)	622 (42.57)	440 (30.12)	1.25 ± 1.16	Low
7. Advise others on choosing medications or health products	256 (17.52)	1019 (69.75)	186 (12.73)	1.22 ± 0.88	Low
8. Use M&HP given by others for trial	196 (13.42)	649 (44.42)	616 (42.16)	0.85 ± 0.97	Low
9. Choose medications with a registration number	71 (4.86)	328 (22.45)	1062 (72.69)	0.37 ± 0.72	Low

Note:.

* The score of usage of M&HP behaviors: ‘always’ (3 points); ‘some time’ (1 point); ‘never’ (0 points), totaling 30 points.

*M&HP Usage Level: ‘Low’: 1.00–1.50, ‘Moderate’: 1.51–2.50, and ‘High’: 2.51–3.00).

The number of respondents for each item may vary due to non-response or item applicability. Analyses were conducted based on available data.

### Factors associated with inappropriate M&HP use

Multivariate logistic regression identified key predictors of inappropriate M&HP use. Rural residence was the strongest factor (adjusted odds ratio [aOR] = 6.33, 95% CI: 2.19–18.31, *p* < .001), followed by limited smartphone literacy (aOR = 4.21, 95% CI: 2.29–7.75, *p* < .001), lower education (aOR = 2.72, 95% CI: 1.48–5.01, *p* = .001), financial insufficiency (aOR = 2.48, 95% CI: 1.40–4.38, *p* = .002), presence of chronic illness (aOR = 1.96, 95% CI: 1.23–3.12, *p* = .004), and unemployment (aOR = 1.85, 95% CI: 1.10–3.09, *p* = .021). These results highlight the multidimensional vulnerabilities influencing unsafe medication practices among rural elderly populations (see Table 4).

**Table 4.** The analysis of the relationship between factors and the usage of M&HP (*N* = 1,461).

Associated Factors	Crude OR (95%CI)	Adjusted OR (95%CI)	P value
**•** Education level lower than primary school	1.52 (1,2.31)	1.82 (1.17,2.81)	.008*
**•** Being married	2.32(1.05,5.26)	2.63(1.16,5.88)	.021*
**•** Unemployment	1.48 (1.18,1.85)	1.49 (1.18,1.88)	< .001**
**•** Living in rural areas	3.49 (1.24,9.82)	6.33 (2.19,18.31)	< .001**
**•** Financial insufficiency	1.33(1.03,1.72)	1.31(1,1.72)	.046*
**•** Having chronic diseases	1.51(1.18,1.96)	1.78(1.37,2.38)	< .001**
**•** Inability to use smartphones effectively	1.56(1.15,2.17)	1.72(1.25,2.38)	< .001**

Note: * Significant (p < 0.05).

** The results of analyzing many factors at a time on the usage of M&HP behavior by multiple logistic regression, backward stepwise: likelihood ratio.

***Wald’s test.

## Discussion

This study examined patterns and determinants of medication and health product (M&HP) use among older adults in rural Northeastern Thailand, revealing a high prevalence of self-medication and reliance on non-professional information sources. These findings align with previous research, which has shown that older adults often lack access to verified medication guidance and face challenges in evaluating product safety, particularly in underserved settings [8,10,23,24].

Despite widespread mobile phone ownership, digital illiteracy remains a significant barrier to safe medication practices. Fewer than 15% of participants accessed health information via smartphones. This reflects the global pattern of digital exclusion among aging populations and supports the WHO’s Global Strategy on Digital Health 2020–2025, which emphasizes inclusive and equitable digital health systems [7,25,26].

Social and economic disadvantages – including rural residency, low educational attainment, financial hardship, and unemployment – were significantly associated with inappropriate M&HP use. These findings reinforce global evidence on the role of social determinants in shaping health behaviors and exacerbating health inequities [27,28]. The presence of chronic conditions may further compound these risks due to increased healthcare needs and reduced self-management capacity in the absence of adequate health literacy support.

The frequent use of dietary supplements, often influenced by cultural beliefs and commercial advertising, poses additional safety and economic concerns [15,29]. However, older adults who remained socially or economically engaged demonstrated better health literacy and safer behaviors, supporting WHO recommendations that active aging and community participation can enhance well-being [30,31].

The reliance on peer and informal networks for health information also highlights the persistent risk of misinformation in this demographic. These results point to the need for health communication strategies that are not only evidence-based but also culturally relevant and accessible to older adults with varying levels of literacy and digital skills [9,32]. While digital literacy and health-seeking behavior were initially considered in the regression analysis, they did not show statistically significant associations with inappropriate M&HP use in this study. Nevertheless, their conceptual relevance remains critical. Digital exclusion and limited help-seeking tendencies may act as upstream barriers, indirectly influencing risky self-care practices through their impact on health information access and decision-making confidence [24–26]. These findings underscore the importance of holistic interventions that improve not only knowledge but also digital access and help-seeking behaviors among older adults [28].

Together, these findings suggest that addressing digital exclusion, strengthening health literacy, and ensuring equitable access to professional healthcare services are essential strategies for mitigating self-medication risks and supporting healthy aging – particularly in rural, low-resource settings standard in LMICs.

### Study strengths and study limitations

This study included a large and stratified sample of older adults from multiple rural communities, enhancing the generalizability of the findings within similar low-resource settings. It provides valuable insights into key risk behaviors, particularly the reliance on non-professional sources for medication advice and limited engagement in digital health information-seeking. These observations align with prior literature on health risks among aging populations in LMICs [8,32,33].

However, the study has limitations. Its cross-sectional nature prevents causal inference, and self-reported data may be influenced by recall or social desirability bias. Furthermore, the findings are based on a Thai context and may not fully reflect health-seeking behaviors in other cultural settings.

This study utilized a large, stratified sample of older adults from diverse rural communities, enhancing the generalizability of findings to similar low-resource settings. It offers insights into key risk behaviors, particularly reliance on non-professional sources for medication and limited use of digital health information. These findings are consistent with prior research on aging populations in low- and middle-income countries (LMICs) [8,32,33].

Several limitations should be noted. First, the cross-sectional design limits causal interpretation, and self-reported data may be subject to recall or social desirability bias. Second, the findings are specific to Thailand and may not be fully generalizable to other cultural contexts.

Third, health literacy was categorized using tertile-based cut-offs due to the lack of a national standard. While common in public health research, this may impact the comparability of results with studies that use standardized tools.

Lastly, digital literacy was measured via a single binary self-report item. Although suitable for low-resource, aging populations, this approach may oversimplify the range of digital skills needed for informed health decisions. Future research should consider multidimensional or performance-based assessments.

## Conclusion

This study highlights the critical roles of health literacy, digital access, and socioeconomic disadvantage in shaping medication behaviors among older adults in rural Thailand. The combined effects of chronic illness, unemployment, and limited education increase vulnerability to inappropriate use of M&HPs. These findings underscore the urgency of context-sensitive interventions that enhance digital health literacy, promote evidence-based self-care, and strengthen local health systems. Ensuring equitable access to reliable health information and professional services is crucial for reducing preventable risks and promoting healthy aging in underserved populations.

## Supplementary Material

Checklist.docx

## Data Availability

The data supporting this study’s findings are not publicly available due to the inclusion of sensitive personal information and restrictions imposed by the consent agreement of participants. Data from the corresponding author may be available upon reasonable request and with appropriate ethical approval.

## Preprint

This article was previously published as a preprint at Research Square: https://www.researchsquare.com/article/rs-6059939/v1
