# Supplementary material for: Health literacy and self-medication among older adults in rural Thailand: understanding inequities in a digital era
Source: Glob Health Action. 2025 Oct 15;18(1):2572008. doi: 10.1080/16549716.2025.2572008 (PMC12529737; doi:10.1080/16549716.2025.2572008)
Supplement: Checklist.docx [file ZGHA_A_2572008_SM8313.docx]

|  | **Standards for Reporting Qualitative Research (SRQR)*** |  |
| --- | --- | --- |
|  | <http://www.equator-network.org/reporting-guidelines/srqr/>  (Note: This SRQR checklist was completed per journal requirement, although the study is quantitative in design.) |  |
|  |  | **Page/line no(s).** |
| **Title and abstract** | |  |
|  | **Title** - Health Literacy and Self-Medication Among Older Adults in Rural Thailand: Understanding Inequities in a Digital Era. | Page 1, Line 1-2 |
|  | **Abstract**: Structured under Background, Objective, Methods, Results, and Conclusion format, per journal guidelines. | Page 1,Lines 17–41 |
|  |  |  |
| **Introduction** | |  |
|  | **Problem formulation**: The study outlines the burden of inappropriate self-medication among older adults in rural Thailand and its relationship to limited health literacy and digital exclusion. | Page 2-4, Lines 44–96 |
|  | **Purpose or research questio**n: To investigate the prevalence and associated factors of inappropriate use of medications and health products among older adults in rural areas of Northeastern Thailand. | Page 4, Lines 92–94 |
|  |  |  |
| **Methods** | |  |
|  | **Qualitative approach and research paradigm**:  Not applicable. This is a quantitative cross-sectional study using structured interviews. | Not applicable |
|  | **Researcher characteristics and reflexivity:**  Researchers included public health and pharmacy professionals. Minimal interaction and objective instruments reduce reflexivity concerns. | Page 1, Lines 5–14 |
|  | **Context** - Setting/site and salient contextual factors; rationale**:  The study was conducted in rural areas of four provinces in Northeastern Thailand—Maha Sarakham, Loei, Nakhon Ratchasima, and Sisaket—which fall under Health Regions 7 to 10. These provinces were selected due to their rapidly aging populations, digital disparities, and socioeconomic vulnerabilities. Elderly schools within these areas were chosen as community-based entry points for participant recruitment. These schools support lifelong learning and community-based health education among older adults. The rationale for selecting this setting was to capture diverse rural perspectives and health behaviors among older adults in LMICs, especially those facing significant barriers in accessing professional healthcare and reliable digital health information. | Page 4, Lines 90–96 |
|  | **Sampling strategy** - Multistage random sampling was employed to ensure representation across diverse rural settings. Elderly schools in four provinces were used as clusters, with proportional selection based on population size. Participants aged 60 years and older were recruited to reflect aging rural communities. The rationale for this strategy was to enhance generalizability across rural Northeastern Thailand. A pre-determined sample size was used based on Cochran's formula, and no further sampling was pursued once the target size was achieved. | Page 5,  Lines 102–110 |
|  | **Ethical issues pertaining to human subjects** - The study protocol was reviewed and approved by the Mahasarakham University Human Research Ethics Committee (Ref. No. 077/2564). All participants provided written or verbal informed consent depending on their literacy and physical abilities. Verbal consent was approved for those who were illiterate or visually impaired. Confidentiality was ensured by anonymizing the data during entry and analysis. Identifiable information was removed and securely stored in a password-protected system, accessible only by the research team. These measures ensured ethical compliance and data protection throughout the study. | Page 6, Lines 145–150 |
|  | **Data collection methods** - Structured interviews were used to ensure consistent data across sites. Data collection was carried out from March to November 2022 using trained health workers. COVID-19 precautions required a mix of face-to-face and remote interviews. While an iterative process or triangulation was not applicable in this quantitative study, procedures were monitored and adapted to ensure completeness and participant safety. The rationale for structured interviews was to obtain standardized responses to support statistical comparison. | Page 6, Lines 135–143 |
|  | **Data collection instruments and technologies** :  The primary instrument used was a structured questionnaire designed to assess demographics, health literacy, and self-medication behavior among older adults. The tool was developed based on a literature review and expert input, and then pre-tested among a similar population. It demonstrated high internal consistency (Cronbach’s alpha = 0.97). Data were collected using printed forms or tablets, depending on local logistics. No significant changes were made to the instrument during the course of the study. Field protocols were standardized to ensure uniformity across data collectors and sites. | Page5, Lines 122-133 |
|  | **Units of study**:  1,461 community-dwelling older adults aged 60 years and over. | Page 5, Lines 106, 106-110 |
|  | **Data processing**:  Data were entered, verified, anonymized, and analyzed using RStudio with descriptive and inferential statistics. | Page 7, Lines 186-188 |
|  | **Data analysis**:  Data were analyzed using descriptive statistics and logistic regression to determine predictors of inappropriate M&HP use. Variables with p < 0.20 from bivariate tests were entered into a multivariable model. RStudio software was used. Although not thematic, this approach enabled empirical inference about population-level associations. The rationale was to identify significant independent predictors to inform public health interventions. | Page 7, Lines 177-185 |
|  | **Techniques to enhance trustworthiness:**  Standardized training and pilot testing ensured the reliability of data collection. Data quality checks were implemented during data entry and cleaning. While methods such as triangulation or member checking were not applicable in this context, audit trails of the data management and analysis process were maintained. The rationale was to enhance reproducibility and minimize bias in a structured quantitative framework. | Page 6, Lines 135-143 |
|  |  |  |
| **Results/findings** | |  |
|  | **Synthesis and interpretation:**  Main findings focused on digital exclusion, health literacy, and predictors of unsafe M&HP use. | Page 8, Lines 177-188 |
|  | **Links to empirical data**:  Supported by statistics, including adjusted odds ratios and 95% confidence intervals. | Page 9-15, Tables and statistical results |
|  |  |  |
| **Discussion** | |  |
|  | **Integration with prior work, implications, transferability, and contribution(s) to the field:**  Connects to global evidence on digital health disparities in aging; relevant for LMIC health systems development. | Page 16, Lines 251–256 |
|  | **Limitations:**  Cross-sectional design limits causality; self-reporting introduces potential biases; findings may be context-specific. | Page 17, Lines 271-276 |
|  |  |  |
| **Other** | |  |
|  | **Conflicts of interest:**  None declared. |  |
|  | **Funding:**  This research project was financially supported by Mahasarakham University. | Page 17, Line 267 |
|  |  |  |
|  | *The authors created the SRQR by searching the literature to identify guidelines, reporting standards, and critical appraisal criteria for qualitative research; reviewing the reference lists of retrieved sources; and contacting experts to gain feedback. The SRQR aims to improve the transparency of all aspects of qualitative research by providing clear standards for reporting qualitative research. |  |
|  |  |  |
|  | **The rationale should briefly discuss the justification for choosing that theory, approach, method, or technique rather than other options available, the assumptions and limitations implicit in those choices, and how those choices influence study conclusions and transferability. As appropriate, the rationale for several items might be discussed together. |  |
|  |  |  |
|  | **Reference:** |  |
|  | O'Brien BC, Harris IB, Beckman TJ, Reed DA, Cook DA. **Standards for reporting qualitative research: a synthesis of recommendations.** *Academic Medicine*, Vol. 89, No. 9 / Sept 2014  DOI: 10.1097/ACM.0000000000000388 |  |
|  |  |  |
|  |  |  |
